# Whey Protein Isolate Microgel Properties Tuned by Crosslinking with Organic Acids to Achieve Stabilization of Pickering Emulsions

**DOI:** 10.3390/foods10061296

**Published:** 2021-06-04

**Authors:** Jéssica Thaís do Prado Silva, João Vitor Munari Benetti, Taís Téo de Barros Alexandrino, Odilio Benedito Garrido Assis, Jolet de Ruiter, Karin Schroën, Vânia Regina Nicoletti

**Affiliations:** 1 Institute of Biosciences, Humanities and Exact Sciences (Ibilce), São Paulo State University, Cristóvão Colombo 2265, São José do Rio Preto 15054-000, SP, Brazil; joao.benetti@unesp.br (J.V.M.B.); vania.nicoletti@unesp.br (V.R.N.); 2 Food Process Engineering Group, Wageningen University & Research, 6700 AA Wageningen, The Netherlands; jolet.deruiter@wur.nl (J.d.R.); karin.schroen@wur.nl (K.S.); 3 Biotechnology Graduate Program (PPG-Biotec), Campus São Carlos (UFSCar), Federal University of São Carlos, Rodovia Washington Luiz km 235, São Carlos 13565-905, SP, Brazil; tais.teo@hotmail.com; 4 National Nanotechnology Laboratory for Agriculture (LNNA), Embrapa Instrumentação, XV de Novembro 1452, São Carlos 13560-970, SP, Brazil; odilio.assis@embrapa.br

**Keywords:** protein microgels, whey protein isolate, particle properties, tannic acid, citric acid

## Abstract

Whey protein isolate (WPI) can be used effectively to produce food-grade particles for stabilizing Pickering emulsions. In the present study, crosslinking of WPI microgels using organic acids (tannic and citric acids) is proposed to improve their functionality in emulsions containing roasted coffee oil. It was demonstrated that crosslinking of WPI by organic acids reduces the microgels’ size from ≈1850 nm to 185 nm and increases their contact angle compared to conventional WPI microgels, achieving values as high as 60°. This led to the higher physical stability of Pickering emulsions: the higher contact angle and smaller particle size of acid-crosslinked microgels contribute to the formation of a thinner layer of particles on the oil/water (O/W) interface that is located mostly in the water phase, thus forming an effective barrier against droplet coalescence. Particularly, emulsions stabilized by tannic acid-crosslinked WPI microgels presented neither creaming nor sedimentation up to 7 days of storage. The present work demonstrates that the functionality of these crosslinked WPI microgels can be tweaked considerably, which is an asset compared to other food-grade particles that mostly need to be used as such to comply with the clean-label policy. In addition, the applications of these particles for an emulsion are much more diverse as of the starting material.

## 1. Introduction

Emulsions stabilized by solid particles (Pickering emulsions) are extensively explored as they offer high stability against coalescence. At the same time, they represent an alternative for avoiding the use of artificial surfactants that, despite obeying government food and health regulations, are less desired from a consumer (clean label) point of view. For these reasons, Pickering emulsions are attractive possible choices for application into foodstuff, whether for designing products that require long shelf life or as a vehicle system for delivery of bioactive compounds [1,2,3].

Current research is focused on the development of Pickering stabilizers using natural and food-grade polymers, such as polysaccharides (starch, cellulose, and chitosan), lipids (fat crystals), and proteins (casein, soybean protein isolate, and zein) [4,5,6]. However, developing food-grade Pickering particles is difficult as they must obey strict prerequisites, such as partial wettability and specific size. Particles should be wetted by both continuous and dispersed phases in order to stay anchored at the interface. This property is related to the three-phase contact angle (θ): if θ << 90°, particles are highly wetted by water, remaining dispersed at the water phase; if θ >> 90°, particles are highly wetted by oil, remaining dispersed at the oil phase. For intermediate θ values, particle adsorption would be optimal with the particles being partially wetted by both phases, which favors embedding in the interface, with very high detachment energy. In addition, large particles could be less efficient in adsorbing at the interface [4,7,8].

Whey protein isolate (WPI) microgels have demonstrated to have strong potential for stabilizing Pickering emulsions, as they comply with the above-mentioned prerequisites and have shown improved functionality as a stabilizer when compared to native WPI [7,8]. Additional strategies have been investigated in order to produce protein microgels of well-defined size and shape and with suitable wetting properties to favor adsorption at interfaces, including jet homogenization, sonication, and enzymatic crosslinking [9,10].

Due to their high nucleophilic amino acid content, proteins are able to engage in various crosslinking reactions. Chemical crosslinking of proteins is mostly performed by reaction with aldehydes, mainly glutaraldehyde and formaldehyde. However, these substances have toxic potential and are not allowed in food [11]; food-grade alternatives are the organic acids tannic acid and citric acid. Tannic acid is a phenolic compound found in many plant sources, which reacts readily with amino acids through its phenolic group, which is an excellent proton donor capable of forming strong hydrogen bonds with the carboxyl groups present in the protein structure [12,13]. Citric acid is a weak organic acid widely used in the food industry as an acidulant and preservative. It is found naturally in citrus fruits, especially in lemon and lime. The carboxylic groups of citric acid react with the free amino groups of the protein to form crosslinks and make citric acid part of the protein network, thus contributing to its hydrophobicity balance [14,15]. Both acids are declared Generally Recognized as Safe (GRAS) by the Food and Drugs Administration (FDA). There are no limitations for adding citric acid into foodstuff, while for tannic acid, the established limit is up to 0.04% [16,17]. Tannic acid is recognized for its antioxidant properties and is widely used as an additive in food products, especially in baking mixes, beverages, and desserts [18,19], in spite of some antinutritional properties (i.e., reduction of protein’s bioavailability during digestion). Tannic acid and citric acid have been demonstrated to improve the technological properties of protein structures, such as macroscopic gels, films, and delivery vehicles [20,21,22,23,24,25]. However, their effect on protein microgel properties and the resulting effects on Pickering emulsion stabilization are yet unknown.

In the present work, the focus is on the production and characterization of food-grade organic acid-crosslinked WPI microgels for applications in oil-in-water Pickering emulsions. The properties of WPI microgels are tweaked by crosslinking with organic acids, and the expectation is that physical properties relevant to their role in Pickering emulsion stabilization will be modified, especially in terms of size distribution, surface charge, and wetting properties. Tannic acid or citric acid is used as the crosslinker and roasted coffee oil as the dispersed phase. This oil is already applied in the food industry as flavoring agent [26], and its use in the present work is proposed as an opportunity to diverse a food material’s application. The produced Pickering emulsions were evaluated for physicochemical characteristics and stability over 7 days of storage. WPI microgels, with and without a crosslinker, were characterized by particle size distribution, zeta potential, and contact angle. Furthermore, Fourier-transform infrared spectroscopy (FTIR), X-ray diffraction (XRD), and scanning electron microscopy (SEM) were performed in order to elucidate the chemical and structural aspects of the particles.

## 2. Materials and Methods

### 2.1. Materials

Whey Protein Isolate (WPI) 88% purity (CL 3987, Alibra Ingredients, Campinas, Brazil) was used to prepare WPI microgels. Tannic acid (99%, Sigma-Aldrich, Darmstadt, Germany—1701.20 g mol^−1^) and citric acid (99%, Synth, Diadema, Brazil—192.12 g mol^−1^) were used as crosslinking agents. Sodium azide (99%, 0.1 g L^−1^, Dinâmica, Indaiatuba, Brazil) was used to prevent microbial growth. Sodium hydroxide and hydrochloric acid solutions (HCl, 0.1 mol L^−1^, Dinâmica, Indaiatuba, Brazil) were used for pH adjustment. Roasted coffee oil was kindly donated by Linax—Essential Oils (Votuporanga, Brazil).

### 2.2. Production of WPI Microgels

WPI microgels were produced according to the methodology described elsewhere [21] with some modifications. In brief, solutions of 40 g L^−1^ WPI in deionized water were prepared, and 0.1 g L^−1^ of sodium azide was added as an antimicrobial agent. WPI solutions were stirred for 2 h at 200 rpm (IKA, HS7, Staufen, Germany) and incubated under refrigeration for 12 h in order to ensure complete protein hydration.

For the production of conventional microgels (WP40), firstly, WPI solutions had their pH adjusted to 5.8 since this pH value has been demonstrated to allow gel structure formation with good sphericity [27]. Then, solutions were heated in a thermal bath at 80 °C for 15 min, after which the samples were quickly cooled to room temperature and sonicated (Sonic Rupter 4000, Omni International, Kennesaw, GA, USA) for 3 min (20 kHz, 400 W), using an ice bath to prevent sample overheating (homogenization temperature was 30 ± 2 °C).

The crosslinker (tannic acid or citric acid) was added to the prepared and cold-incubated WPI solutions (see the previous description). From preliminary studies, we selected to work at 3:1 (crosslinker:protein) molar ratio based on the protein content in solution (the purity of WPI was taken into account, and the molar mass of the major constituent (β-lactoglobulin) was used as that of the protein material). After crosslinker addition, the pH was adjusted to 5.8, and the same procedure—as described above for conventional WPI microgels—was followed.

### 2.3. Characterization of WPI Microgels

#### 2.3.1. Particle Size Distribution and Zeta Potential

Particle size distribution and zeta potential were measured through dynamic light scattering (DLS) and electrophoretic light scattering, respectively. Samples were diluted 1000× in deionized water and evaluated (Zetasizer Nano ZS, Malvern Panalytical, Malvern, UK) immediately after their production. Each sample was analyzed in triplicate at 25 °C, using 1.45 as material refraction index for the protein particles, 1.33 as dispersant (water) refraction index, and 0.8872 mPa s as dispersant viscosity.

#### 2.3.2. Fourier-Transform Infrared Spectroscopy (FTIR)

The chemical structures of conventional and crosslinked WPI microgels were investigated by attenuated total reflection Fourier-Transform Infrared spectra (ATR-FTIR). The spectra were recorded (Vertex 70 Spectrometer, Bruker Corporation, Berlin, Germany) in absorbance mode in a spectral region of 4000—480 cm^−1^, over 64 consecutive scans. Prior to analysis, the microgel dispersions were frozen in ultra-freezer at −35 °C (Liotop, Liobras, São Carlos, Brazil) right after preparation and subsequently freeze-dried for 24 h (L101, Liobras, São Carlos, Brazil).

#### 2.3.3. X-ray Diffraction (XRD)

X-ray patterns of freeze-dried samples, including native WPI, were obtained in an X-ray diffractometer (Miniflex 300, Rigaku, Japan). The samples were held on a glass support and subjected to scans from 3 to 40° (2θ) at a rate of 1° min^−1^; using Cu Kα radiation generated at 30 kV and 10 mA. The crystallinity indices (Crl) were calculated according to the Segal method (Equation (1)) [28], where $I_{\max}$ is the maximum intensity (height of the tallest peak in the diffractogram), and $I_{\mathrm{am}}$ is the intensity corresponding to the amorphous fractions of the samples.

(1) $$\mathrm{Crl}\;\left(\%\right)=\left[\left(I_{\max}-I_{\mathrm{am}}\right)/I_{\max}\right]\times 100$$

#### 2.3.4. Scanning Electron Microscopy (SEM)

Microgel morphology was characterized by means of scanning electron microscopy with a field emission gun (XL-30 FEG, Philips). Before analysis, the samples were frozen at −35 °C for 12 h followed by drying for 24 h using a freeze dryer (Liotop L101, Liobras, Brazil). The freeze-dried samples were then diluted in deionized water, fixed on carbon tape, and metallized with gold. Images were acquired at magnifications of 2000×, 5000×, and 20,000×, with electron beam acceleration of 5.0 kV.

#### 2.3.5. Contact Angle

For static contact angle measurements, the microgel dispersions were carefully pipetted on microscopy slides. The slides were put in a desiccator to evaporate water so that only the deposited microgels remained on the glass surface, forming a thin layer. These slides were then placed in a contact angle measurement system (CAM 101, KSV Instruments, Helsinki, Finland) with a coupled camera, and one drop of deionized water (2 µL) was deposited on each microgel film [29]. The experiment was performed in triplicate, and images were acquired immediately after the water drop was deposited on the microgel film.

### 2.4. Production of Pickering Emulsions Containing Roasted Coffee Oil

Microgel dispersions prepared with 40 g L^−1^ of WPI as described in Section 2.2., with and without crosslinker, were used as the continuous phase for preparing Pickering emulsions containing roasted coffee oil as the dispersed phase (10% *w/w*). As a blank, an emulsion prepared with a solution of native WPI (40 g L^−1^) was used. The roasted coffee oil and the continuous phase were pre-homogenized using Ultra Turrax (T-25, IKA, Staufen, Germany) for 2 min at 9000 rpm. Then, the coarse emulsions were sonicated (Sonic Rupter 4000, Omni International, Kennesaw, GA, USA) for 3 min (20 kHz, 400 W) while immersed in an ice bath to prevent overheating (homogenization temperature was 30 ± 2 °C).

### 2.5. Characterization of Pickering Emulsions Containing Roasted Coffee Oil

#### 2.5.1. Morphology and Droplet Size

Images of the emulsion droplets were obtained using a bright-field microscope (CX31, Olympus, Tokyo, Japan) with a 100× magnification lens, with a coupled camera (SC30, Olympus). Around 20 µL of the emulsion was pipetted on a glass slide and coated with a coverslip, followed by adding one droplet of immersion oil. These microscopy images were evaluated using ImageJ (NIH) software to determine the diameter of 300 droplets in each sample, and frequency distribution graphs were made using the software GraphPad Prism v. 8 (San Diego, CA, USA), which enabled identification of D_10_, D_50_, and D_90_ for each distribution. The distribution width (Span) was calculated according to Equation (2), in which $D_{i}$ is the diameter, below which i% of the sample is contained (i= 10, 50, 90) [30,31].

(2) $$\mathrm{Span}=\left(D_{90}-D_{10}\right)/D_{50}$$

#### 2.5.2. Evaluation of Emulsion Stability

Physical stability of the produced emulsions was evaluated by the creaming (CI) and sedimentation (SI) indexes. For this purpose, emulsions were transferred to test tubes in triplicate and stored for 7 days at room temperature. The CI was calculated according to Equation (3), where $H_{U}$ is the height of the upper phase (lowest density), and $H_{T}$ is the total height of the emulsion in the tube [32].

(3) $$\mathrm{CI}\;\left(\%\right)=\left(H_{U}/H_{T}\right)\times 100$$

Similarly, the sedimentation index was calculated using Equation (4), where $H_{S}$ is the sediment height in the test tube.

(4) $$\mathrm{SI}\;\left(\%\right)=\left(H_{S}/H_{T}\right)\times 100$$

### 2.6. Statistical Analysis

The results of analytical determinations for WPI microgels (particle size, polydispersity index, and zeta potential) and Pickering emulsions (creaming and sedimentation indexes) were subjected to one-way analysis of variance (ANOVA). Samples were evaluated in triplicate, and statistical analyses were carried out using the software Statistica v. 12.0 (StatSoft, Hamburg, Germany), considering a significance level (*p*) of 0.05 and Tukey’s test as post hoc.

## 3. Results and Discussion

### 3.1. Characterization of WPI Microgels

Particles used for Pickering stabilization must meet specific criteria related to size, charge, morphology, and partial wettability to accomplish a high performance as an emulsion stabilizer. In this section, these characteristics of the produced WPI microgels were assessed. The influence of organic acids during the crosslinking step was investigated, and hypotheses about the potential of the various microgels for Pickering stabilization were formulated, which will be experimentally studied in the next section.

#### 3.1.1. Size and Zeta Potential

Particle size distributions of the produced microgels are summarized in Table 1, which shows that smaller and less polydisperse particles could be attained when a crosslinker was used. The DLS measurement of WP40 points to large particles (Table 1) and highly polydisperse size distribution, probably indicating that conventional microgels are forming aggregates. In fact, sedimentation can be observed within a few minutes, which can be related to gravitational separation due to the microgels’ size and density difference with the continuous phase (water). The crosslinked microgels were 8–10 times smaller than the conventional microgels and did not present sedimentation.

Farjami et al. [21] stated that the crosslinker increases the structural stability of proteins during heat treatment, preventing agglomeration and thus resulting in relatively smaller particles when compared to microgels produced in the absence of crosslinker. These authors produced WPI microgels crosslinked by citric acid with sizes ranging from 80 to 130 nm, which are smaller than those obtained in the present work, although in a similar order of magnitude. The size difference may be the result of a different WPI source and ratio crosslinker: protein but was most probably caused by the method applied for size distribution analysis: atomic force microscopy, which is determined by a scanning probe instead of light scattering. Crosslinking with tannic acid yielded the smallest microgels and lowest polydispersity index (PDI) among all treatments. In fact, WPTA was the only treatment that resulted in PDI lower than 0.2, which is the limit generally used for monodisperse polymer-based nanoparticles [33].

The zeta potential values of the microgels in suspension were negative for all the samples (Table 1). The absolute values as measured for WPI microgel complexes are dependent on the protein concentration and can vary from positive to negative according to the acidity of the medium [34,35]. Values close to −30 mV are commonly found in the literature for WPI based microgels [36] and even lower values [37], with reasonable stability of the particles in suspension. The isoelectric point of WPI is stated as pH 5.0, and the dissociation of COOH groups above pH 5 led the particles to carry negative charges [29]. It is worth mentioning that in the present study, the microgels were processed at pH 5.8, with general results in good agreement with cited literature.

#### 3.1.2. Fourier-Transform Infrared Spectroscopy (FTIR)

**Figure 1:**
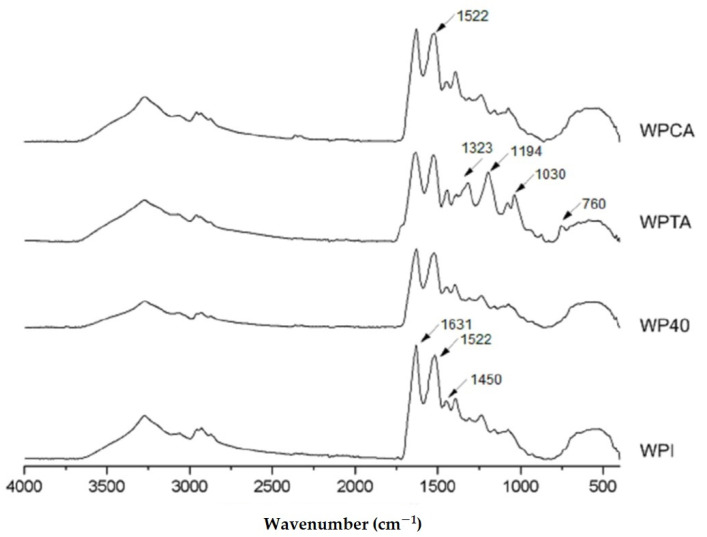
Infrared spectra of native whey protein isolate (WPI), conventional microgels (WP40), tannic acid- (WPTA), and citric acid-crosslinked (WPCA) WPI microgels.

Figure 1 presents the ATR-FTIR spectra of native WPI, WP40, WPTA, and WPCA. All samples have similar fingerprints related to the specific functional groups of WPI.

The two main peaks at 1631 cm^−1^ and 1522 cm^−1^ relate to peptide bonds of amide I (C=O stretching) and amide II groups (N–H folding and C–N stretching), respectively, which are very characteristic of protein structures. A peak centered at 1450 cm^−1^, with lower intensity, corresponds to C–N stretching and N–H folding of the amide III group [38]. The amide I band (1631 cm^−1^) gives information about the protein secondary structure; more specifically, the bands at around 1650–1660 cm^−1^ represent α-helix structures, whereas bands between 1610–1640 cm^−1^ refer to β-sheet structures [9]. The heating of WPI solutions leads to partial modification of the secondary structure [39], as can be observed by comparing the WPI and WP40 spectra (Figure 1). The band related to the secondary structure (1631 cm^−1^) underwent intensity reduction after heat treatment (sample WP40), indicating partial unfolding or loss of some of the protein original helix configuration.

The effect of crosslinking can be also observed, mainly for tannic acid. Two features are important to be highlighted: a new band appears at 1030 cm^−1^ in the WPTA spectrum, which is related to C–O stretching vibration in the amide III region [11], whereas the peaks assigned at around 1323 cm^−1^ and 1194 cm^−1^ in the WPTA spectra correspond to the most strong absorptions identified in the tannic acid spectra (not shown), and are related to the Csp2–O bond of the acid aromatic rings [40]. These bands are not observed in the protein isolate structure. Thus, the presence in the WPTA spectra indicates the incorporation of the tannic acid in the microgel formation. Most of the interactions between TA and proteins are described as occurring in the amide regions involving the participation of TA phenolic hydroxyls and the amides in the proteins (CN and NH group). These interactions probably predominate hydrogen bonding rather than ionic crosslinking between species [40,41,42]. Additionally, the simultaneous occurrence of hydrophobic interactions is considered a possible cooperative mechanism in the formation of protein-tannin complexes [43,44,45].

The spectrum of WPCA microgels is mostly unchanged compared to WPI, indicating that the general structure of proteins is preserved. However, there is an increase in peak intensity in the amide II region (1522 cm^−1^), which is likely due to the occurrence of interactions between C–N groups. This is consistent with the observation by Farjami et al. [21], who reported chemical linking between the carbonyl groups of citric acid and the amino groups of proteins.

**Figure 2:**
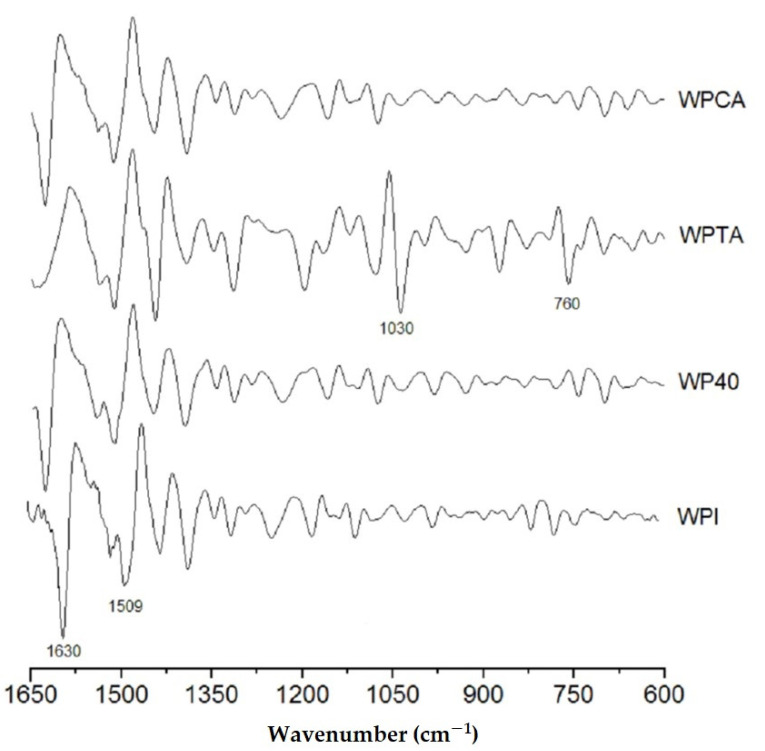
The second derivative of the original FTIR spectra of native WPI, conventional microgels (WP40), tannic acid- (WPTA), and citric acid-crosslinked (WPCA) WPI microgels.

Figure 2 shows the second derivative of the original spectra, which permits a much more detailed qualitative analysis. This evaluation focused mainly on the region between 1650 and 600 cm^−1^, which comprises the amide I, amide II, and amide III structures of the whey protein. The smoothed derivative peaks follow a Lorentzian function shape, as well known from literature [46].

In the control WPI, two main peaks are worth signaling: the stronger band at 1630 cm^−1^, related to β-segments, and a weaker one centered at 1509 cm^−1^ that is associated with the α-helix conformation. The peak corresponding to β-structure almost vanished in the tannic acid crosslinked sample, reflecting the strong interaction between TA and WPI that promotes a denaturation of secondary β-conformation protein structure, while the α-helix conformation is preserved.

It is evident that WPTA resulted in a structure that most differs from the original WPI control. Two more intense peaks appear at 1030 and 760 cm^−1^. The first is ascribed to intense absorbance from C–O stretching and N–H deformation in the amide III groups [11]. The second less intense band could be related to the side-chain vibrations from the whey protein isolate [47], which probably reflects the tertiary structure associated with hydrogen bonding reaction between the amide and hydroxyl groups of the tannic acid. 

#### 3.1.3. X-ray Diffraction (XRD)

**Figure 3:**
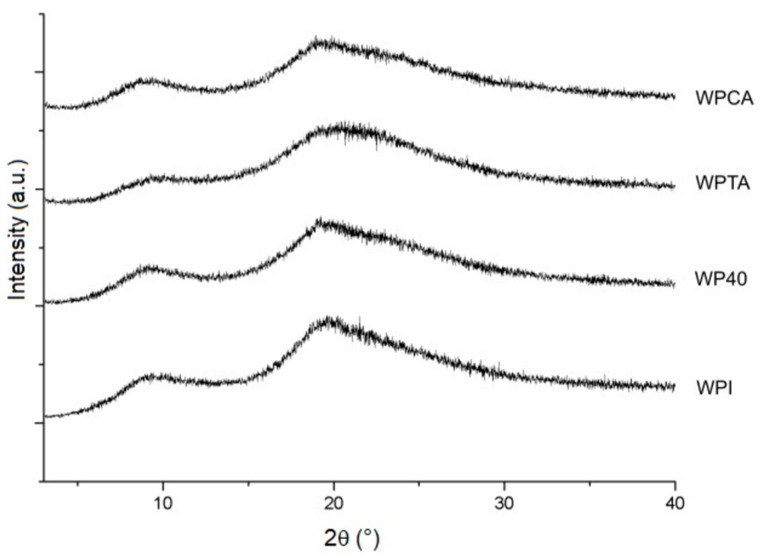
X-ray diffraction patterns of native WPI, conventional microgels (WP40), tannic acid- (WPTA), and citric acid-crosslinked (WPCA) WPI microgels.

The diffractograms presented in Figure 3 confirm the semi-crystalline nature of WPI and the microgels. All samples show similar patterns with main diffraction peaks related to α-helix (9.2°) and β-sheet (19.6°) structures [48,49]. A crystallinity index of 58.6% was calculated for WPI, with a small decrease upon processing, except for the WPCA sample that retained the same crystallinity index as WPI (Table 2), as was also expected based on the FTIR result presented earlier. Similar results were reported by Mohammadian et al. [50] and Sun et al. [49] for conventional WPI microgels prepared at different pH values.

In spite of the crystallinity indexes measured being a result of the drying procedure necessary to the analysis, the differences between samples may be related to the organization degree of protein structure when dispersed in the aqueous medium. The degree of WPI crystallization, as obtained in dry conditions, reflects the limited mobility and rotations of the groups in the protein structure. In wet conditions, the access of water molecules to the hydrophilic groups is easier in a less crystalline structure, thus facilitating nucleation and more uniform size distribution of the microgels. The crosslinking with tannic acid resulted in a structure with lower crystallinity (Table 2). This justifies, to some degree, the average particle size and polydispersity values as displayed in Table 1.

#### 3.1.4. Scanning Electron Microscopy with Field Emission Gun (SEM-FEG)

**Figure 4:**
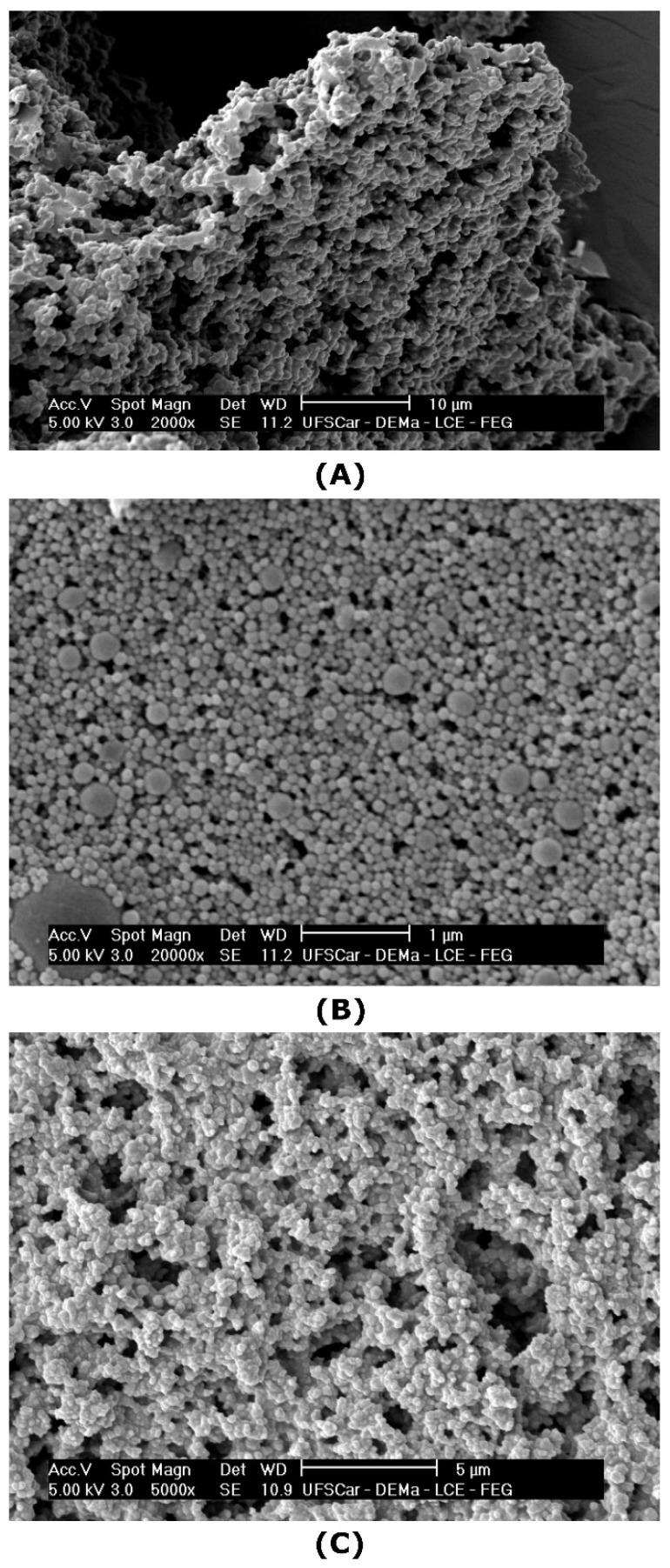
SEM images of: (**A**) conventional microgels (WP40) (2000×); (**B**) tannic acid- (WPTA) (20,000×); (**C**) citric acid-crosslinked (WPCA) (5000×) WPI microgels.

SEM images of the microgels are presented in Figure 4. After freeze-drying and redispersion in water, conventional microgels remained aggregated (Figure 4A), forming a spongy structure, as reported for WPI microgels by other researchers [51]. Conversely, the nanoparticles crosslinked with tannic acid, sample WPTA (Figure 4B), were small and with a nearly spherical shape, with a more homogenous distribution, revealing a lower tendency to form aggregates. The citric acid crosslinked nanoparticles (Figure 4C) have some similarities with WPTA microgels concerning size, although assumed a configuration of irregular aggregates when dried under microscopic observation.

In Pickering emulsions, particle shape is an important parameter for emulsion stability as it will determine how particles are going to pack themselves at the interface [52]. As outlined by Berton-Carabin and Schroën [4], food-grade particles are very complex and may have irregular shapes; however, often their structure is soft, which allows for the particles to flatten at the O/W interface, resulting in efficient interfacial anchorage and possibly also network-formation, which contributes to emulsion stability. If this applies to the conventional and crosslinked microgels produced in this work, this may render them suitable for Pickering stabilization, as reported later.

#### 3.1.5. Contact Angle

The contact angle measurements are an indication of the usefulness of particles for Pickering stabilization. Particles that are totally hydrophilic (contact angle θ = 0°) will remain in the water phase, and particles that are highly hydrophobic (θ = 180°) would remain in the oil phase [53]. For intermediate angles, the particle can be positioned at the O/W interface, although the effect on emulsion stability can vary greatly [54].

**Figure 5:**
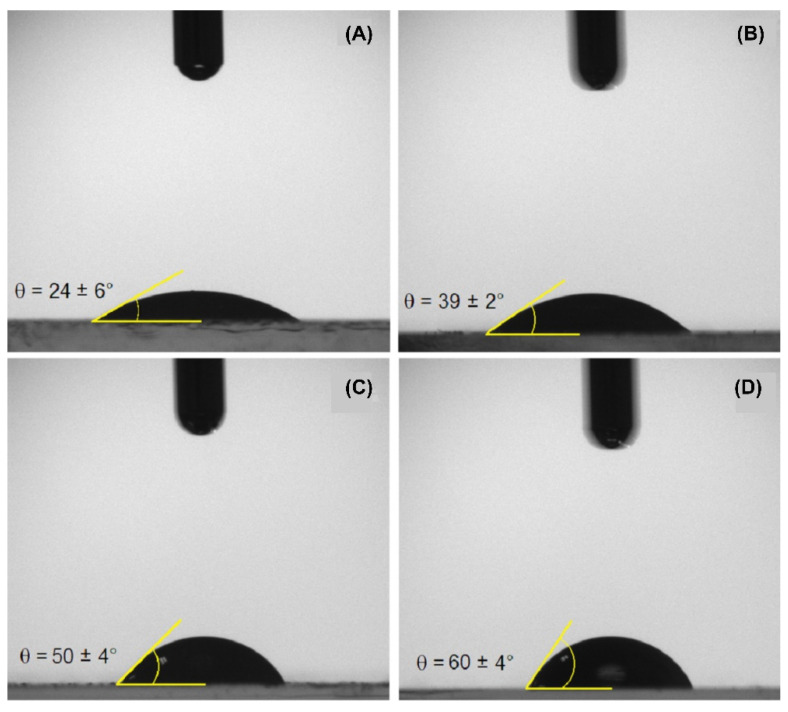
The water contact angle (in air) on: (**A**) native WPI; (**B**) conventional microgels (WP40); (**C**) tannic acid-crosslinked (WPTA); (**D**) citric acid-crosslinked (WPCA) WPI microgels.

In the present study, the water contact angle measurements of the solid particles were performed in the air (θ_sw(a)_) by gently depositing a water droplet (2 µL) onto the layer of dried particles under ambient atmosphere. It is important to clarify that the exact value of the (water) contact angle of a single microgel particle at the O/W interface will differ from that measured on a dried film surface (θ_sw(a)_) for two main reasons: first, because of the different ambient phase, and second, due to the differences between the microscopic contact on a single (swollen) particle, and the macroscopic contact angle measured on a grafted microgel film [55]. However, the macroscopic contact angle in the air (see Figure 5) does show a strong correlation with the microscopic contact angle, which allows for the identification of trends in particle wettability, as was already discussed by Wu et al. [29].

WPI consists of a group of globular proteins that, in their native state, present their hydrophilic fractions oriented outward, while hydrophobic groups are folded inside the molecule [56]. For this reason, depositing a water droplet onto the WPI film (Figure 5A) results in the smallest contact angle among the treatments, 24 ± 6°, confirming the surface predominance of hydrophilic groups. Particular attention was given to minimize the effect of protein dissolution by taking the measurement around 1 second after droplet deposition. For WPI microgels (Figure 5B), higher contact angles were found as for WPI, which can be indicative of exposure of hydrophobic groups as a result of previously discussed structural changes.

Both crosslinked particles (Figure 5C,D) show considerably higher contact angles than their non-crosslinked counterparts (Figure 5B), which is indicative of higher hydrophobicity. The polarity change may have occurred via chemical bonding between crosslinkers and carboxyl groups or amines in the protein structure, consequently reducing the number of polar side groups available to interact with water molecules [57]. The contact angle increased from 39 ± 2° for WP40 to a maximum of 60 ± 4° for WPCA, which will allow these particles to attach more strongly to the emulsion interface compared to conventional microgel particles.

### 3.2. Characterization of Pickering Emulsions Containing Roasted Coffee Oil

Pickering emulsions were prepared using the produced WPI microgels as stabilizers and ultrasound as the dispersion method. In this section, the emulsions containing roasted coffee oil are evaluated regarding droplet size, morphology, and stability. This oil is an important byproduct of the coffee industry, and it has been applied as a flavoring agent, especially for baking products. Since Brazil is the largest coffee producer in the world, it is desirable to develop new technologies for expanding the use of coffee byproducts [58]. We would like to stress that WPI microgels can stabilize emulsions with other, more conventional, disperse phases, and demonstrated this for hexadecane. In this way, here, we focus on roasted coffee oil as a way to indicate the versatility of such emulsions in terms of application in the food industry.

#### 3.2.1. Morphology and Droplet Size

**Figure 6:**
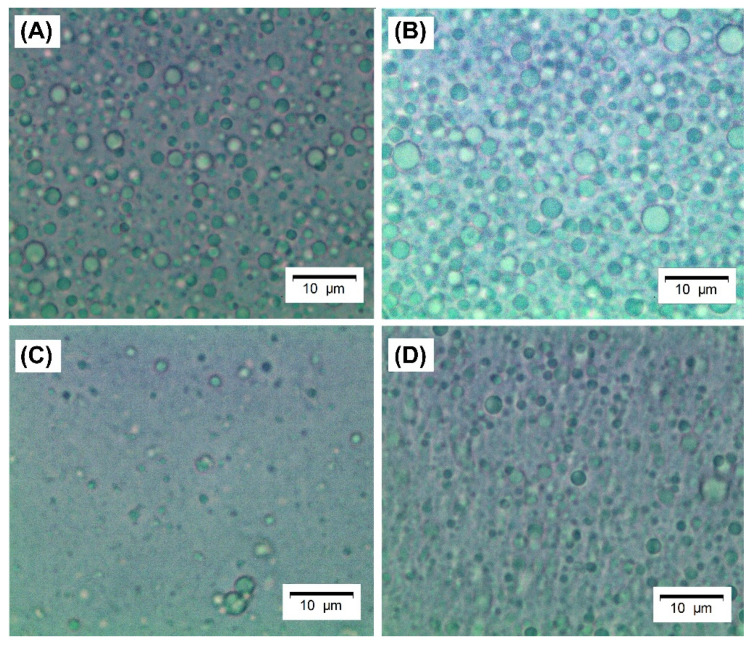
Light microscopy images of emulsions stabilized by: (**A**) native WPI solution 40 mg mL^−1^; (**B**) conventional microgels (WP40); (**C**) tannic acid-crosslinked (WPTA); (**D**) citric acid-crosslinked (WPCA) WPI microgels. Scale bars refer to 10 µm.

Images of the produced emulsions were obtained by bright-field microscopy and are shown in Figure 6. The emulsions stabilized by crosslinked microgels (Figure 6C,D) have slightly smaller droplets, as shown in Table 3, which seem not to be flocculated, unlike the emulsion stabilized by regular WPI and WPI microgels (Figure 6A,B).

In emulsions formulated with WPI and WP40, destabilization took place after 12 h, probably due to droplet flocculation and further coalescence, generating creaming effects (see also Table 4). It is important to point out that it is unlikely that the large aggregates of WP40 microgels (see Table 1) can stabilize an emulsion with the average droplet size of 1.96 µm since it is necessary that particles are at least 10-fold smaller than the droplet size [4]. However, it is likely the large aggregates of conventional microgels were disintegrated during high-pressure homogenization. The size of the (clustered) droplets is, of course, the most important reason for droplet creaming as, in accordance with Stokes’ law [59,60], the particle radius has a squared effect on the upward (or downward) velocity of the dispersed phase. On the other hand, the crosslinked microgels might have resulted in a higher packing density at the interface that, in turn, may have reduced the density difference between the dispersed and continuous phase; another possible effect is that the more rigid crosslinked particles were less prone to form bridged interfaces that favors flocculation [61]; both effects reduce gravitational separation.

#### 3.2.2. Physical Stability of Pickering Emulsions

The emulsion stabilized with the microgels crosslinked with tannic acid is the only one that is stable to phase separation (Table 4) and shows creaming and sedimentation indexes of 0 after 7 days of storage. All other emulsions showed considerable creaming and, in two cases, also sedimentation.

The emulsion stabilized by native WPI did not show any sedimentation, which is logical since WPI is a soluble small molecule that, when dispersed properly, does not sediment. The emulsion stabilized by WPCA showed some sedimentation, indicating that these microgels are slightly prone to gravitational separation, albeit not as much as the standard microgels that are 10-fold larger in size (Table 1). The hypothesis is that the sedimented fraction is constituted by protein particles that were not adsorbed at the oil/water interface. This phenomenon was previously observed by de Folter et al. [62] and Anjali and Basavaraj [63] for hematite regular-shaped particles but also for food-grade particles based on WPI and soluble soybean polysaccharides, as reported by Cabezas and et al. [64]. These authors observed that the denatured protein particles with larger sizes (around 240 nm) would be less prone to efficiently accommodate at the oil/water interface. In the present work, the samples in which there was sedimentation were those formulated with WPCA (around 258 nm) and WP40 (around 1853 nm). The appearance of sedimented particles also helps to explain the higher creaming indexes observed in these emulsions, as the less effective particle adsorption at the interface resulted in larger droplets, more susceptible to coalescence and further creaming. Ding and et al. [65] produced Pickering emulsions stabilized by glutaraldehyde-crosslinked gelatin nanoparticles that were not that effective compared to the crosslinked WPI microgels used in the present study.

## 4. Conclusions

Crosslinking of WPI microgels with organic acids improves their physical and chemical characteristics by yielding particles that are smaller, less polydisperse, and more hydrophobic than conventional WPI microgels, which contribute to more effective adsorption at the O/W interface during the formation of Pickering emulsions. In particular, tannic acid demonstrated to be a very suitable WPI crosslinker for providing emulsions with higher stability. This is in stark contrast to emulsions stabilized by native WPI or conventional WPI microgels, in which droplet flocculation and coalescence were observed within a few minutes after emulsification. Even though the adsorption dynamics of WPI microgels is still unknown, the results presented in this work support the hypothesis that crosslinking of WPI microgels by organic acids, such as tannic acid and citric acid that are recognized as GRAS, is a promising strategy for producing crosslinked WPI microgels with tailored functionality for Pickering stabilization. However, the amount of emulsions stabilized by tannic acid-crosslinked microgels into foodstuff should be selected according to recommendations of regulatory agencies. On the other hand, the emulsification process with WPI microgels (with or without crosslinking) needs to be better understood. Residual soluble proteins may compete with microgels in stabilizing the O/W interface and thus influence the emulsion formation and/or stability. Our future research will be focused on a deeper understanding of the formation of crosslinked WPI microgels, including the fraction of proteins that remain in solution. It is also important to address the residual amount of crosslinking agent that carries astringency and thus may reduce consumer acceptance due to sensory inappropriateness. If this is the case, an additional step would need to be added to the production line of the microgels, and although that is technically very feasible, it will add to the overall costs. To be complete, the stability of the emulsions under processing conditions will be to be checked.

## Figures and Tables

**Table 1:** Particle size, polydispersity index (PDI), and zeta potential of conventional microgels (WP40), tannic acid- (WPTA), and citric acid-crosslinked (WPCA) WPI microgels.

Sample	Particle Size (nm)	PDI	Zeta Potential (mV)
WP40	1852.6 ± 638.0 ^a^*	0.981 ± 0.03 ^a^	−32.1 ± 0.3 ^ab^
WPTA	185.2 ± 2.2 ^b^	0.123 ± 0.01 ^c^	−33.4 ± 1.1 ^a^
WPCA	257.6 ± 4.2 ^b^	0.212 ± 0.01 ^b^	−30.3 ± 0.5 ^b^

Different letters in the same column indicate significant difference according to Tukey’s mean test. Data expressed as mean (*n* = 3) ± standard deviation. * Analysis without quality criteria due to high polydispersity.

**Table 2:** Crystallinity indices (Crl) of native WPI conventional microgels (WP40), tannic acid- (WPTA), and citric acid-crosslinked (WPCA) WPI microgels.

Sample	Crl (%)
WPI	58.6
WP40	53.9
WPTA	52.1
WPCA	60.0

**Table 3:** The mean droplet size (D_A_) and polydispersity (span) of emulsions containing roasted coffee oil stabilized by native WPI, conventional microgels (WP40), tannic acid- (WPTA), and citric acid-crosslinked (WPCA) WPI microgels.

Continuous Phase	D_A_ (µm) *	Span	D_10_ (µm)	D_50_ (µm)	D_90_ (µm)
WPI	1.80	0.96	0.93	1.79	2.64
WP40	1.96	0.95	1.12	1.86	2.88
WPTA	1.26	0.98	0.75	1.15	1.88
WPCA	1.54	1.22	0.81	1.35	2.46

* Corresponds to the arithmetic mean of the diameter of 300 droplets in each sample.

**Table 4:** Creaming and sedimentation indexes of Pickering emulsions containing roasted coffee oil stabilized by native WPI, conventional microgels (WP40), tannic acid- (WPTA), and citric acid-crosslinked (WPCA) WPI microgels.

Continuous Phase	Creaming Index (%)	Sedimentation Index (%)
WPI	4.0 ± 0.5 ^a^	0.0 ± 0.0 ^c^
WP40	4.4 ± 0.1 ^a^	10.4 ± 0.4 ^a^
WPTA	0.0 ± 0.0 ^b^	0.0 ± 0.0 ^c^
WPCA	3.8 ± 0.5 ^a^	2.4 ± 0.1 ^b^

Different letters in the same column indicate significant difference according to Tukey’s mean test. Data expressed as mean (*n* = 3) ± standard deviation.

## Data Availability

Not Applicable.
